# Confirmation of ovarian follicles in an enantiornithine (Aves) from the Jehol biota using soft tissue analyses

**DOI:** 10.1038/s42003-020-01131-9

**Published:** 2020-07-28

**Authors:** Alida M. Bailleul, Jingmai O’Connor, Zhiheng Li, Qian Wu, Tao Zhao, Mario A. Martinez Monleon, Min Wang, Xiaoting Zheng

**Affiliations:** 1 Key Laboratory of Vertebrate Evolution and Human Origins, Institute of Vertebrate Paleontology and Paleoanthropology, Chinese Academy of Sciences, 100044 Beijing, China; 2 CAS Center for Excellence in Life and Paleoenvironment, 100044 Beijing, China; 3 State Key Laboratory of Palaeobiology and Stratigraphy, Nanjing Institute of Geology and Paleontology, Chinese Academy of Sciences, 210008 Nanjing, China; 4 Department of Ecology, Faculty of Science, Charles University, Prague, CZ-12844 Czech Republic; 5 Institute of Geology and Paleontology, Linyi University, 276005 Linyi City, Shandong China; 6 Shandong Tianyu Museum of Nature, 273300 Linyi City, Shandong China

**Keywords:** Palaeontology, Biogeochemistry

## Abstract

The remains of ovarian follicles reported in nine specimens of basal birds represents one of the most remarkable examples of soft-tissue preservation in the Early Cretaceous Jehol Biota. This discovery was immediately contested and the structures alternatively interpreted as ingested seeds. Fragments of the purported follicles preserved in an enantiornithine (STM10-12) were extracted and subjected to multiple high-resolution analyses. The structures in STM10-12 possess the histological and histochemical characteristics of smooth muscles fibers intertwined together with collagen fibers, resembling the contractile structure in the perifollicular membrane (PFM) of living birds. Fossilized blood vessels, very abundant in extant PFMs, are also preserved. Energy Dispersive Spectroscopy shows the preserved tissues primarily underwent alumino-silicification, with minor mineralization via iron oxides. No evidence of plant tissue was found. These results confirm the original interpretation as follicles within the left ovary, supporting the interpretation that the right ovary was functionally lost early in avian evolution.

## Abstract (web summary)

Bailleul et al. employ histology, histochemistry and Energy Dispersive Spectroscopy to confirm the presence of disputed ovarian follicles in a specimen of fossil Cretaceous bird. These findings have implications for the evolution of the avian breeding system seen in birds today.

## Introduction

Since the discovery of the first feathered birds and non-avian dinosaurs from the Jehol Biota of northeastern China in the 1990s, thousands of specimens have been reported^1^. These volcanolacustrine deposits^2^ represent a taphonomic window through which exceptionally well-preserved specimens have revealed important aspects of the biology of a diversity of organisms also including salamanders, pterosaurs, fish, and mammals^1,3,4^. Specimens are typically more than 90% complete, fully articulated and often preserving soft tissues^1^. In addition to feathers, soft tissue remnants reported from Jehol birds include skin^5^, cartilage and ligaments^6^, lungs^7^, and perhaps most controversial of all, ovarian follicles^8,9^.

These follicles were described in nine specimens of early birds, including one specimen of the basal, long bony-tailed *Jeholornis*, one specimen of the confuciusornithiform *Eoconfuciusornis*, and seven specimens of enantiornithines, the dominant clade of land birds during the Cretaceous^5,8–10^. More precisely, the follicles were interpreted as remnants of the perifollicular membrane (PFM) of pre-ovulatory ovarian follicles, a connective tissue that surrounds the yolk-filled oocytes prior to ovulation in extant birds^9^. In all specimens, the purported follicles are preserved as agglomerated, circular, or oval structures on the left side of the body cavity, which led to the conclusion that the earliest birds were like their extant relatives in having only one functional ovary (the left)^8,9^. Together with information from an oviraptorosaur with two eggs preserved dorsal to its pubic symphysis^11^, this suggested that the loss of function of the right ovary occurred very close to the dinosaur–bird transition^9^ supporting existing hypotheses that this loss was linked to the evolution of flight (i.e., being supposedly aerodynamically advantageous, in that the loss of one ovary would have reduced mass and facilitated flight during reproductive periods^12^). Additionally, because all purported follicles are approximately the same (relative) size in all specimens, it was hypothesized that basal birds had a low degree of follicular hierarchy due to slow yolk deposition reflecting their relatively lower basal metabolic rate^8^, which is unlike the condition in modern birds in which the follicular hierarchy is pronounced. This inference was supported by the discovery of the follicles preserved in *Eoconfuciusornis*, which showed a stronger hierarchy than that in *Jeholornis* and enantiornithines, consistent with interpretations that confuciusornithiforms had relatively elevated metabolic rates when compared to other basal avian lineages^5^.

The identification of fossilized ovarian follicles in Jehol birds has been controversial^13–17^. Because follicles are soft tissue structures (i.e., originally unbiomineralized) mostly consisting of viscous yolk surrounded by a thin PFM, it was argued that they are unlikely to fossilize and an alternative hypothesis was proposed, interpreting these structures as ingested seeds (i.e., stomach contents, or cololites)^13–15^. The original identification of the follicles and the secondarily proposed alternative hypothesis were both based entirely on macroscopic morphological observations and preserved anatomical location.

These two conflicting hypotheses can easily be tested at the tissue level. If the purported follicles are indeed remnants of a PFM, they should present the same histological characteristics as the homologous tissues forming the PFM in the ovaries of extant, reproductively active birds. The PFM (or theca) surrounds the yolk-filled oocytes and links them to the ovarian medulla^18^. The three main components of a PFM are collagen fibers, smooth muscle fibers, and blood vessels^18–21^. On the other hand, the tissues forming seeds (or any type of plant material) are completely different and their cells possess a distinct cell wall absent in all animal cells^22^.

To investigate these competing hypotheses, we conducted an in-depth microscopic analysis of fragments extracted from the purported follicles preserved in an enantiornithine from the Shandong Tianyu Museum of Nature, STM10–12, originally described by O’Connor et al.^8^. We used standard ground-sectioning methods, scanning-electron microscopy (SEM), and paraffin histology conducted on demineralized samples to compare the morphology and histochemistry of the fossilized structures with tissues composing pre-ovulatory follicles in an extant hen (*Gallus gallus domesticus*; the extant homologue for this study), and to published data on seed tissues^22^. Histological observations were paired with energy-dispersive spectroscopy (EDS) in order to further understand the chemical alterations that these fossilized soft-tissues underwent during diagenesis. Our results do not show any evidence of plant tissues, but instead reveal histological and histochemical data consistent with fossilized smooth muscles fibers, collagen fibers, and blood vessels. Together, these fossilized tissues show the same organization and characteristics of the contractile structure that expels the oocyte during ovulation in living birds^19,20^. This study confirms both the original interpretation as follicles within the left ovary, and that the right ovary was functionally lost early in avian evolution^8,9^.

## Results

### Surface texture

**Fig. 1:**
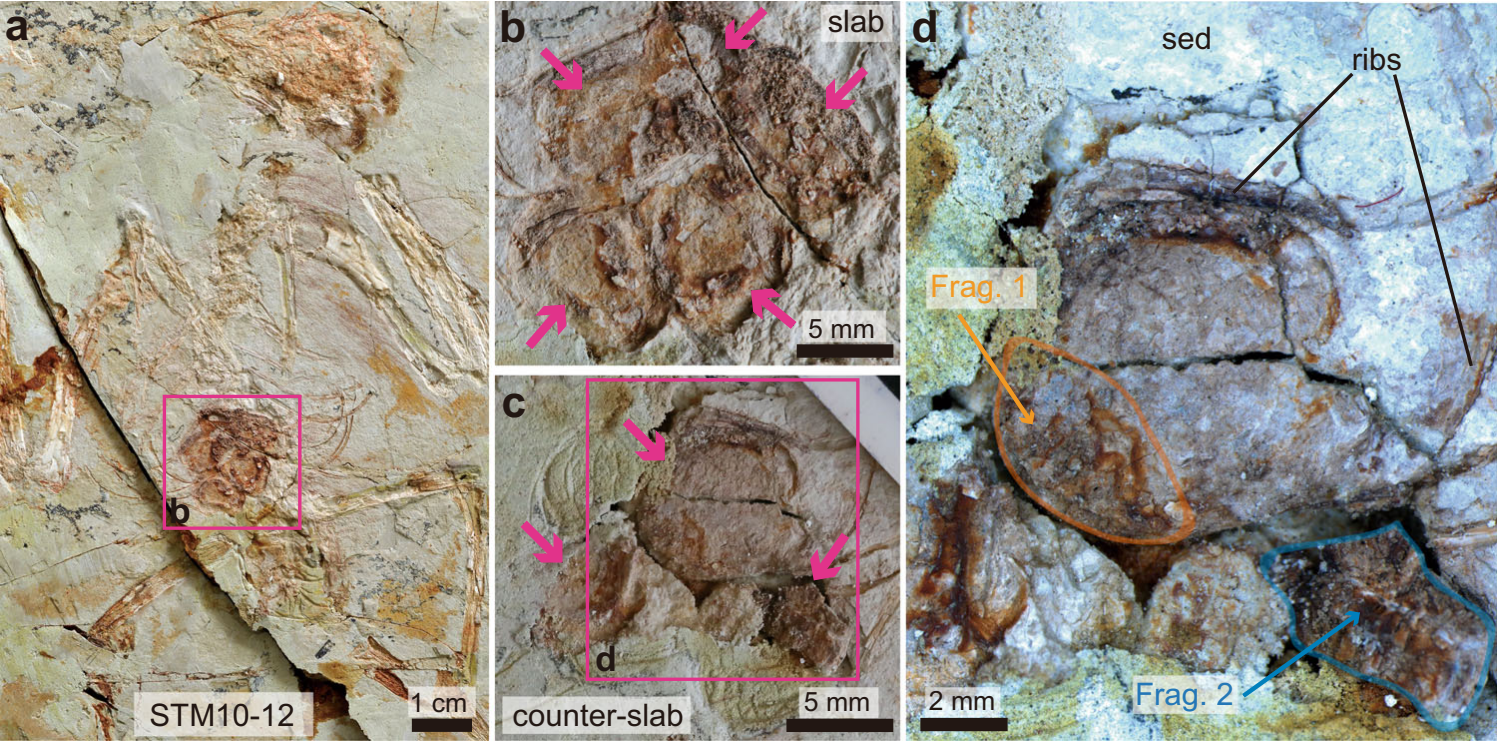
Photograph of an enantiornithine indet, STM10–12. General view of the slab **a** and close-up on the purported ovarian follicles, all approximately the same size (pink arrows in **b**). Close-ups on these same structures (pink arrows) in the counterslab **c**. Two fragments were used for different analytical methods **d**. The first fragment (orange outline) was prepared using ground sectioning methods. The second fragment (blue outline) was demineralized and processed for paraffin histology. Abbreviations: Frag. fragment, sed sediment.

The ovarian follicles reported in the nine basal bird specimens all differ in gross morphology and surface texture (summarized in Supplementary Table 1). In STM10–12 (Fig. 1a) at least five circular structures are visible in the slab (Fig. 1b), and at least three are visible in the counterslab (Fig. 1c). These traces consist of pale pink impressions within a lighter sediment. They are organized in clusters similarly to follicles in extant birds, but unlike the modern avian condition (Supplementary Fig. 1a) they all have approximately the same size (average diameter of 7.7 mm; Fig. 1b, c). Some areas show a cracked surface, with a brown or dark orange tint (Fig. 1d). Two fragments were extracted from the counterslab and used for tissue analysis (Fig. 1d). Prior to being embedded and sectioned, the surface of the first fragment (Fragment 1; Fig. 1d) was observed under the SEM, which revealed it is coated in a thick layer of glue/consolidant, which was applied during fossil preparation prior to this study (Supplementary Fig. 2).

### Histology and histochemistry

**Fig. 2:**
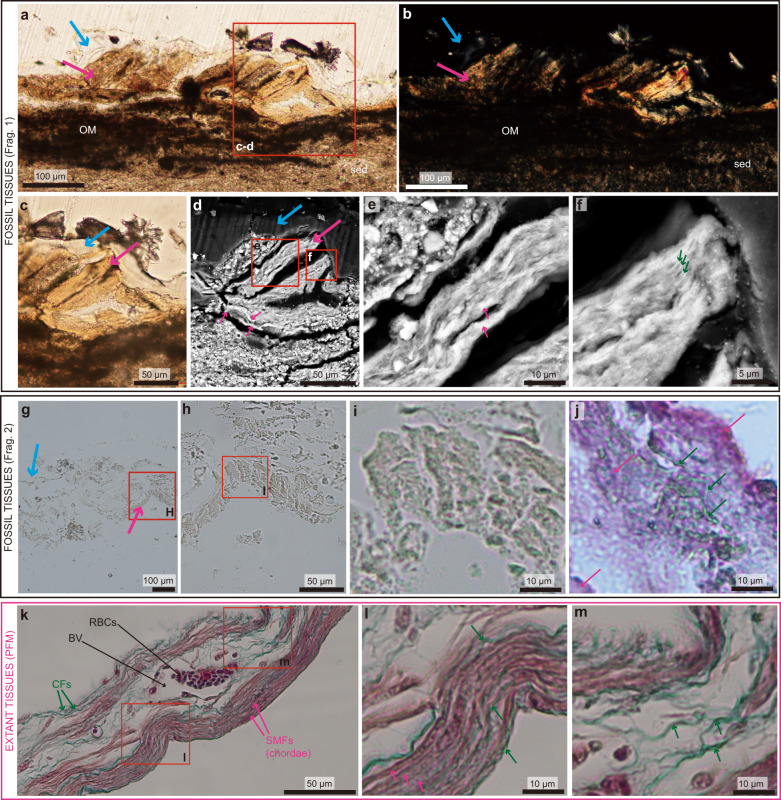
Microscopic analyses of STM10-12 reveal structures consistent with collagen fibers and smooth muscle fibers. Ground-section images **a**–**c**, SEM images **d**–**f** and demineralized paraffin slides of STM10–12 **g**–**j** reveal structures consistent with extant collagen fibers and smooth muscle fibers **k**, **l**. **a** A cross section of the first fragment seen under transmitted light shows a transparent layer of consolidant (blue arrow), and a brown material underneath it representing the preserved soft-tissues (pink arrow). **b** Same image under the polarized light. **c** Close-up on the consolidant and the brown material. **d** Complementary SEM image, showing fibers in the brown material (pink arrows). **e** Close-up showing undulating, thick fibers, consistent in size with smooth muscle fibers. **f** Another close-up showing much thinner fibers between 0.3 and 1 µm thick, consistent with the size of collagen fibers. **g** Unstained paraffin slide of the second fragment of STM10–12, showing a fibrous material (pink arrow), covered by glue (blue arrow). **h** Close-up of red square in **g**. **i** Close-up of red square in **h**. **j** Adjacent slide of STM10–12 stained with Masson’s trichrome. **k** Extant PFM tissues from an ovarian follicle near ovulation (extant hen) stained with Masson’s trichrome. It shows smooth muscle fibers (SMFs, pink arrows) and collagen fibers (CFs; green arrows) organized into a chordae, and some blood vessels. **l** Close-up on the lower red square in **k**. **m** Close-up on the upper red square in **k** showing some CFs with their typical crimp-waveform arrangement. The fossil material shows the same morphology and staining pattern as extant SMFs and CFs organized into a chordae. Images **e**, **i**, **j**, **l**, **m** are shown at the same scale for direct comparison. Additional abbreviations: BV blood vessels; OM organic matter, RBCs red blood cells, sed sediment.

General overview: A ground section of the first fragment (Fragment 1; Fig. 1d) shows three main characteristics: (1) the above-mentioned top layer of consolidant (blue arrow); (2) a light brown material below it (pink arrow); and (3) a bottom layer of sediment (Fig. 2a). The consolidant is transparent and between 20 and 50 µm thick. It is covered by crystals in a few areas (Fig. 2a), and faintly birefringent under polarized light (Fig. 2b). Right below it, the brown material does not possess any clearly identifiable tissue structures nor cells, even when observed at high magnification under transmitted light (Fig. 2c). Under polarized light, this brown material is strongly birefringent (Fig. 2b). However, SEM revealed that this material is composed of fibers (compare Fig. 1c, d). Right below this fibrous material the top layer of the sediment contains a black tint resembling decayed organic matter (Fig. 2a, b).

Fossilized fibrous tissues: When the fibrous material is observed at high resolution under the SEM, densely packed, undulating fibers of two different size ranges can be seen (Fig. 2e, f): larger fibers with a thickness between 2 and 3 µm (Fig. 2e), and smaller fibers between 0.3 and 1 µm thick (Fig. 2f).

The second sample (Fragment 2; Fig. 1d) was demineralized and paraffin sections were made at 5 µm (Fig. 2g–j). The demineralized sample revealed densely packed, undulating fibrous material (pink arrow; Fig. 2g–i).

Morphological and histochemical comparison with extant fibrous tissues: We compared the fossilized undulating fibers seen in STM10–12 to the fibers composing the PFM of an ovarian follicle near ovulation from an extant hen (Fig. 2k–m). The extant PFM was stained with a modified Masson’s trichrome (a common connective tissue stain^23^), revealing blood vessels surrounded by a connective tissue made of collagen fibers (stained green) and larger smooth muscle fibers (stained pink) (Fig. 2k–m). This fibrous structure, called the chordae, contracts to expel the yolk-filled oocyte during ovulation^19–21^ (Fig. 2k). Collagen fibers are easily recognizable by their small size and their wavy organization, referred to as a crimp waveform arrangement (e.g., ref. ^24^; Fig. 2l, m).

At low magnification, the overall shape and undulating pattern of the fossilized fibrous material is consistent in size and morphology to the chordae in our extant *Gallus* sample (compare Fig.  2h with Fig. 2k). The two different size ranges of fibers seen in the fossil under SEM (Fig. 2e, f) are also consistent with the size of the smooth muscle fibers and collagen fibers in the extant material (Fig. 2l, m). When stained with Masson’s trichrome (Fig. 2j), the fossilized fibers also showed a staining pattern very similar to that seen in the extant chordae (compare Fig. 2j, l, m, shown at the same scale). Most of the fossilized fibrous material stained pink, and a few smaller fibers stained green and showed the typical crimp waveform arrangement unique to collagen fibers (Fig. 2j).

**Fig. 3:**
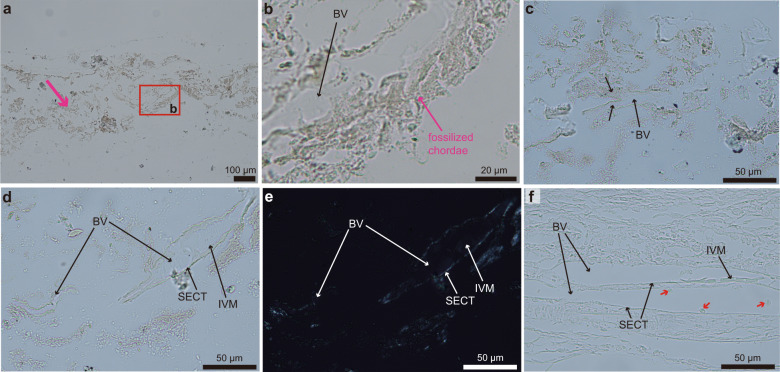
Demineralized paraffin slides of STM10–12 reveal structures consistent with extant blood vessels. **a** Unstained slide of STM10–12 showing the preserved fibrous soft-tissues (pink arrow). **b** Close-up showing a structure resembling a blood vessel (BV) near the fossilized chordae. **c** Close-up in another slide showing bifurcation (black arrows) in a BV-like structure. **d** Close-up from another unstained slide showing BV-like structures with walls and internal material. **e** Corresponding image under the polarized light. **f** Unstained PFM from an extant hen showing blood vessels, red blood cells (red arrows), their sub-endothelial connective tissue (SECT) and intravascular material (IVM). Images **d**–**f** are shown at the same scale for direct comparison.

Fossilized vessel-like structures and comparison with extant vessels: Additional structures were observed in the paraffin slides made from the demineralized sample of STM10–12, which were not seen in the ground-section nor the SEM images. Near the fossilized chordae some hollow, tubular structures can be seen (Fig. 3a–e). These structures sometimes show a branching pattern (Fig. 3c) and have walls (Fig. 3d, e). These structures present the same morphological characteristics of extant, avian blood vessels (Fig. 3f). The size of these vessel-like structures falls within the size range of extant blood vessels observed in an extant PFM (e.g., compare Fig. 3d with Fig.  3f, shown at the same scale).

The walls of the fossilized blood vessels are birefringent under polarized light (Fig. 3d) and are morphologically similar to the sub-endothelial connective tissue observed in extant blood vessels (Fig. 3f). The inside of the tubular vessels preserves an amorphous substance that resembles intravascular material (IVM) sometimes visible in histological sections of extant blood vessels (compare the IVM in Fig. 3d with Fig. 3f), most likely representing fossilized blood breakdown products and/or plasma (e.g., see Fig. 1h in Schweitzer et al.^25^).

**Fig. 4:**
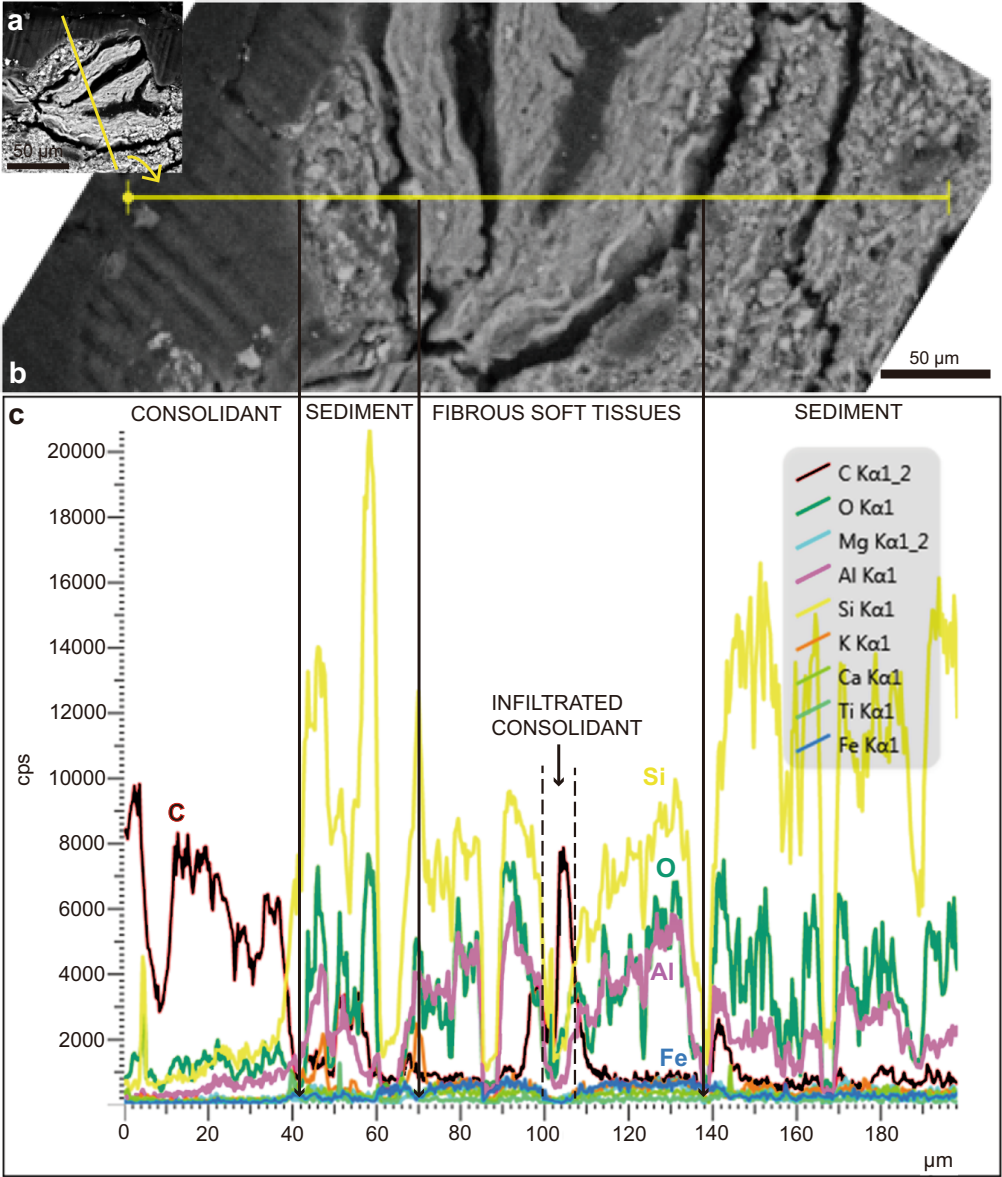
EDS profile on the preserved soft tissues of STM10–12 shows they underwent alumino-silicification and slight iron mineralization. **a** SEM image (same image as Fig. 2d) with yellow line showing where the EDS profile was taken. **b** Enlargement and horizontalization of **a**. The EDS profile was followed along the external layer of glue, the soft tissue layer with preserved fibers, and the sediments below them. **c** EDS profile showing the chemistry of all features (glue, soft tissues, and sediments) of major elements in counts per second (cps). The layer of glue is highly enriched in carbon (C), followed by low amounts of oxygen (O), silicon (Si) and aluminum (Al). The Si and Al signals seen in the glue are most likely a contaminant from the sediment and/or soft tissues beneath. The Si signal may also come from the glass of the ground-section. The soft tissues (concluded to be part of the original perifollicular membrane) beneath the consolidant show a net drop in C content, but a clear increase in Si, O, and Al. The source of Al in the soft-tissues may come from clay minerals within the sediments below, and it is possible that direct precipitation of authigenic clays onto the tissues occurred shortly after death. The soft-tissues are also enriched in Iron (Fe, blue line) when compared to the sediments. There are also negligible amounts of magnesium (Mg), potassium (K), calcium (Ca), and titanium (Ti) in the sample.

Energy-dispersive spectroscopy: The soft tissues observed in the ground-section (i.e., the fossilized chordae with blood vessels) were chemically analyzed. EDS shows the preserved soft-tissues are enriched in silicon, oxygen, aluminum, with low amounts of carbon and iron. Compared to the sediment there is a net enrichment in aluminum and iron in the soft-tissues (Fig. 4). These results suggest that during diagenesis the soft-tissues primarily underwent alumino-silicification, with slight mineralization via iron oxides (Fig. 4). The chemical signal of the consolidant is very different from the soft-tissues and consists mostly of carbon and oxygen (Fig. 4).

## Discussion

When first described, it was hypothesized that the circular traces preserved in basal birds from the Jehol Biota represented remnants of the PFM of mature or nearly mature follicles within the left ovary^9^. The absence of calcified eggshell in the oviduct suggested that ovulation had not begun in any specimen. In one enantiornithine (STM29-8), the unusual surface texture in the purported follicles was interpreted as the imprints of a well-developed network of blood vessels within a highly vascularized PFM^9^ (Supplementary Fig. 3).

The results of our analyses support identification of the remains preserved in enantiornithine STM10–12 as remnants of the ovary and a vascularized PFM. The tissues in STM10–12 present the same morphological and histochemical characteristics as those of an avian chordae, a contractile structure made of intertwined collagen fibers with smooth muscle fibers that expel the oocyte during ovulation in extant birds (Fig. 2). STM10–12 also preserves structures morphologically consistent with extant blood vessels (Fig. 3). Given that the analyzed fragments of purported follicles in STM10–12 present virtually all the tissue characteristics (i.e., appropriate size, morphology, and histochemistry) of the three main components found in extant PFMs (i.e., smooth muscle fibers, collagen fibers, and blood vessels) the most parsimonious and plausible conclusion is that the circular structures in STM10–12 are indeed the fossilized remnants of pre-ovulatory ovarian follicles, also consistent with their preserved anatomical location. Fossilized structures morphologically consistent with collagen fibers and muscle fibers have been previously identified in numerous other Mesozoic specimens (e.g., refs. ^26–31^). This study contributes to the mounting evidence that such tissue components can preserve in deep-time.

The fossilized follicles in STM10–12 are by no means completely preserved, but rather represent fragments of these structures. We found no histological evidence of other tissues that are found in the PFM of extant pre-ovulatory follicles, such as non-collagenous fibers of the inner perivitelline membrane, granulosa cells, nerve fibers^32^, and the ovarian surface epithelium (Supplementary Fig. 1).

Fossilized blood vessels have been previously reported in some specimens of Mesozoic dinosaurs and Cenozoic turtles (e.g., refs. ^33–36^). The vessels in STM10–12 are not consistent in morphology with fungal hyphae in that they lack septae and fungal hyphae are much smaller (see refs. ^33,34^). Given their size and morphology, the most logical interpretation is that these structures are remnants of original blood vessels belonging to an originally highly vascularized PFM.

The fossilization of blood vessels is apparently more common than generally recognized in material from the Mesozoic and these structures have already been thoroughly documented both morphologically and chemically (e.g., refs. ^26,29,33,34,37–39^). In previous studies, fossil blood vessels were mostly observed in three dimensions (3D), photographed as ‘floating’ material in demineralizing solutions, whereas in this study the blood vessels were observed in 2D sections exposing the blood vessels through longitudinal cuts (Fig. 3). In blood vessels analyzed in 3D, branching is also commonly observed (e.g., see refs. ^33,38,40^) but in contrast, in sectioned vessels branching is much less common (e.g., see Fig. 3f–h), which may explain why very few branching patterns were observed in our sample (only in Fig. 3c).

Noteworthy, blood vessels in STM10–12 were only visible in the demineralized paraffin slides (Fig. 3) but not in the ground-sections nor the SEM images (Fig. 2). A possible logical explanation is that this is due to differences in tissue compaction and distortion between the samples in the ground-sections and the paraffin slides. In the ground sections, the tissues were embedded in resin without being demineralized. Resin-embedded tissues are tightly compacted (Fig. 2d) and do not undergo any significant distortion during preparation. On the other hand, demineralized tissues that get embedded in paraffin go through multiple distortion and tearing events (i.e., during demineralization, processing through different solutions, but especially after being cut on a microtome and placed on top of warm water in the water bath prior to mounting on glass slides, see Supplementary Methods). These distortions create an artificial ‘decompaction’ of the fossil tissues in the paraffin slides (which occurs commonly even while making slides of extant tissues), and this is most likely what enabled the visualization of blood vessels in STM10–12 (Figs. 2g–j and 3) that were not directly visible in ground-sections nor through SEM (Fig. 2d). These results suggest that the three methods employed here (ground-sectioning, SEM, and paraffin histology) yield complementary information and, when used together, can help to provide more rigorous identifications and clarify our understanding of fossilized soft-tissues.

EDS showed that the fossilized soft-tissues preserved in STM10–12 underwent alumino-silicification (Fig. 4). This same process has been reported in fossilized branchiopod (clam shrimp) eggs also from the Jehol Biota, where all the envelopes were made of calcium phosphate but some of the eggs had their internal contents replaced by alumino-silicates^41^. An explanation for the mechanism of alumino-silicate replacement was not provided for the Jehol clam shrimp eggs^41^, but we suggest that clay minerals from sediments were involved. Clay minerals have been determined to be important agents in the fossilization of soft-tissues in other settings (such as the Ordovician Soom Shale of South Africa and the Cambrian Burgess Shale of Canada)^42,43^. In these cases, the soft-tissues were replaced rapidly after death by authigenic clay minerals (via direct precipitation of authigenic clays onto the tissues). It is the most logical explanation for the process of alumino-silicification seen in the tissues of STM10–12, and this process may have happened rapidly after death before extensive tissue decay.

EDS also revealed an enrichment in iron, indicating that the soft-tissues may also have experienced some limited mineralization with iron oxides (Fig. 4, see the low iron levels). The mineralization of soft-tissues via iron oxides (such as goethite and biogenic iron oxyhydroxide) has been reported in Mesozoic dinosaurs^38^ and a similar process may have occurred as well in STM10–12. A potential source for this iron may be the pyroclastic flows that intermittently interrupted the deposition of lacustrine sediments, and/or microbial mats, as proposed for Jehol invertebrates^44,45^. Moreover, based on our new data, we also suggest that another source of this iron may be the hemoglobin (a protein found in red blood cells) coming directly from the blood vessels of the organism itself. An experimental study on ostrich blood vessels demonstrated that iron and oxygen from hemoglobin play a key role in tissue stability and in the exceptional preservation of soft-tissues in deep-time^38^. This hypothesis could be tested in the future with immunohistochemistry and antibodies raised against avian hemoglobin.

Although pyritization (via iron sulfides) has been demonstrated in the soft-body parts of Jehol insects and hypothesized to play an important role in the preservation of soft-tissues in Jehol fossils^44,45^, the EDS data here do not show any sulfur in the sample, meaning STM10–12 underwent a different fossilization process and that pyritization is simply one of the many processes involved in soft-tissue preservation in the Jehol paleolakes.

All of the histological and histochemical data collected here (Figs. 1–4) demonstrate the exceptional preservation of the soft-tissues in STM10–12 (i.e., fossilized chordae and blood vessels from the PFM), but more precise and more specific chemical analyses (such as synchrotron-FTIR, or immunohistochemistry) are needed to fully characterize the preservation of these tissues at a deeper molecular level.

Until this study, interpretations of the purported follicles as ingested seeds (e.g., see Supplementary Fig. 4) indeed represented a viable alternative hypothesis^13,14^. Testing these competing hypotheses was imperative to our understanding of both the evolution of the paravian reproductive system (i.e., to confirm whether or not early birds indeed had only one functional ovary like extant birds and lacked strong follicular hierarchy) and digestive system (as yet there is no direct evidence regarding the diet of enantiornithines in the Jehol^46,47^).

Many morphological arguments have previously been raised against the interpretation of these remains as ingested seeds^5^. For example, the preserved structures also lack the surficial ornamentation observed in most fossilized seeds (Supplementary Fig. 4). Furthermore, the tissues here identified in STM10–12 (smooth muscle fibers, collagen fibers, and blood vessels; Figs. 2 and 3) are strictly animal tissues and are non-existent in plants. None of the histological slides of STM10–12 reveal tissues reminiscent of fossilized plant material with their characteristic cell walls^22,48^, from either gymnosperms or angiosperm seed or fruit tissues (e.g., cuticle, seed coat or testa, internal integument layers, and embryonic tissues; e.g., refs. ^22,49,50^). Although our original intent was to directly compare the tissues in STM10–12 with a fossil seed preserved in the stomach of the holotype of *Jeholornis prima* (Supplementary Fig. 4), close examination showed that the seeds in this specimen are only impressions, and thus cannot be used for proper comparison with seed tissues. Lastly, the STM10–12 samples were also checked for the presence of phytoliths, which are microscopic structures made of silica that are found in plant tissues and can persist for millions of years after the decay of the plant^51^, but none were found.

Although the preservation of ovarian follicles in STM10–12 is confirmed, this hypothesis needs to be independently tested in each individual specimen (Supplementary Table 1); as yet, it is still possible some of the purported follicles in other specimens may represent ingested seeds. Confirmation of follicle preservation in one specimen does nonetheless put an end to the controversy regarding whether or not such remains can preserve and confirms that only a single functional ovary was present in at least some non-neornithine avians^8,9^. Additionally, our results support paleobiological hypotheses based upon the original identification, such as observations regarding the relative sizes of these ovarian follicles. Compared to modern birds only subtle size variations are observed in the fossilized follicles of Jehol birds (meaning follicular hierarchy was absent), leading to the inference that in basal birds yolk deposition occurred much more slowly than in extant bird due to the lower metabolic rates of non-ornithuromorph birds^8,9^. This study reveals that this was true at least for enantiornithine STM10–12, which suggests this may be similarly true about other non-ornithuromorph birds in which slow growth rates are observed through osteohistology. It is likely that a strong follicular hierarchy is a derived feature of a subset of the Ornithuromorpha (because some of them still retain plesiomorphically slower growth rates), but only direct evidence with preserved follicles in Cretaceous ornithuromorphs could confirm this hypothesis.

The Early Cretaceous Jehol Biota of China preserves one of most extraordinary extinct fauna and flora ever discovered, revealed through a taphonomic environment that was extremely conducive to the fossilization of both hard and soft-tissues^1,4^. Few histological analyses on preserved soft-tissues exist due to the destructive nature of most of these methods. However, our results demonstrate that these types of analyses can eliminate doubt and help to further understand preservation. Previous taphonomic studies conducted on Jehol invertebrates showed that alumino-silicification and pyritization help preserve soft-tissues in the Jehol^41,45^. In STM10–12, no pyritization was found, but the tissues apparently underwent alumino-silicification and a slight mineralization with iron, potentially iron oxides. This suggests that soft-tissue fossilization in the Jehol is case-specific and that varied mechanisms were involved. In the case of animal tissues, it is possible that preservation of soft tissues was facilitated by endogenous iron and oxygen from the hemoglobin in blood^26,38^. The abundant blood vessels in the PFM may explain the high preservation potential of ovarian follicles, although this hypothesis cannot explain why other tissues and organs that are also highly vascularized are not preserved in the same specimens.

In STM10–12 and all other specimens with purported follicles, the overall spherical shape of the follicles is preserved in 2D. Therefore, it is also possible that organic remnants from the original spherical yolk and/or the inner perivitelline membrane are preserved, but extensive further analyses (using different methods such as immunohistochemistry or spectroscopy) are required to confirm this. Additionally, the most external tissue covering preovulatory follicles in extant birds is made of pancytokeratin^18^, a hydrophobic protein with a high potential for fossilization^52^. We propose that this hydrophobic molecule may have acted as a barrier and also facilitated the exceptional preservation of follicles. Much more research is necessary to confirm this hypothesis, and more studies on preserved soft-tissues in the Jehol are necessary to further shed light on the modes of tissue preservation through deep-time.

## Methods

### Ground-sectioning

The two fragments from the counterslab of STM10–12 were extracted from the fossil with a scalpel. One sample was observed under the SEM (Supplementary Fig. 2) and then embedded in EXAKT Technovit 7200 (Norderstedt, Germany) one-component resin, and cured for 24 h, cut using an EXAKT 300CP accurate circular saw, and then ground and polished using the EXAKT 400CS grinding system (Norderstedt, Germany) until the desired optical contrast was reached (about 50 μm). Sections were observed under transmitted and polarized light using a Nikon eclipse LV100NPOL, and photographed with a DS-Fi3 camera and the software NIS-Element v4.60. Paraffin sections (see below) were observed and photographed with this same system.

### SEM-EDS

The ground-section was analyzed using SEM at the Chinese Academy of Geological Sciences with a FEI Quanta 450 (FEG) at 20 kV (Fig. 2). Both BSE and SE modes (backscattered electrons and secondary electrons) were applied. The EDS profile were measured as a line, continuous between different layers (Fig. 4).

### Paraffin histology

A few extant samples of whole ovarian follicles (‘large white follicles’) and the PFM of a more mature follicle (‘hierarchical follicle’) completely filled with yolk were dissected from a gravid female chicken obtained commercially (Supplementary Fig. 1). These extant tissues were fixed in 10% neutral buffered formalin (NBF) for 48 h. Demineralization of the extant tissues was not necessary as they are all unbiomineralized soft tissues. The second fragment from STM10–12 was embedded in 3% agar (Becton Dickinson Cat# 214530) (for stabilization) and demineralized (without NBF) in 500 mM EDTA; pH 8.0 for 3 weeks with a solution changes every 2–3 days. Demineralization of the fossil tissues was made simply to comply with the microtome blades and paraffin protocols. All tissues (extant and fossils) were subjected to routine dehydration, to clearing in xylene, and to paraffin infiltration and embedding (Supplementary Methods). Sections were cut at 5 µm on a rotary microtome (Leica Biosystems RM2265), placed into a warm water bath (at about 44 °C) and mounted on charged slides (Superfrost Plus, Fisher Scientific). Most slides of STM10–12 were left unstained: they were simply deparaffinized in different solutions of xylene for about 15 min and cover-slipped with mounting medium (Permount, Fisher Scientific). Some slides (of STM 10–12 and extant tissues) were stained with a modified Masson’s trichrome^23^ (Supplementary Methods). Contamination between fossil and extant tissues has been avoided (see Supplementary Methods).

### Reporting summary

Further information on research design is available in the Nature Research Reporting Summary linked to this article.

## Supplementary Information

Supplementary Information

Reporting Summary

## Data Availability

STM10–12 is reposited at the Shandong Tianyu Museum of Nature in Linyi City. Ground-sections and paraffin sections of STM10–12 fragments are currently reposited at the Institute of Vertebrate Paleontology and Paleoanthropology in Beijing. All data are available upon reasonable request.
