# Supplementary material for: Confirmation of ovarian follicles in an enantiornithine (Aves) from the Jehol biota using soft tissue analyses
Source: Commun Biol. 2020 Jul 28;3:399. doi: 10.1038/s42003-020-01131-9 (PMC7387556; doi:10.1038/s42003-020-01131-9)
Supplement: Supplementary file 1 — Supplementary Information [file 42003_2020_1131_MOESM1_ESM.pdf]

Supplementary Information

Confirmation of ovarian follicles in an enantiornithine (Aves) from the Jehol biota through soft tissue identification

Bailleul et al.

This PDF file includes:

Supplementary Figures 1 to 4

Supplementary Table 1

Supplementary Methods

Supplementary References

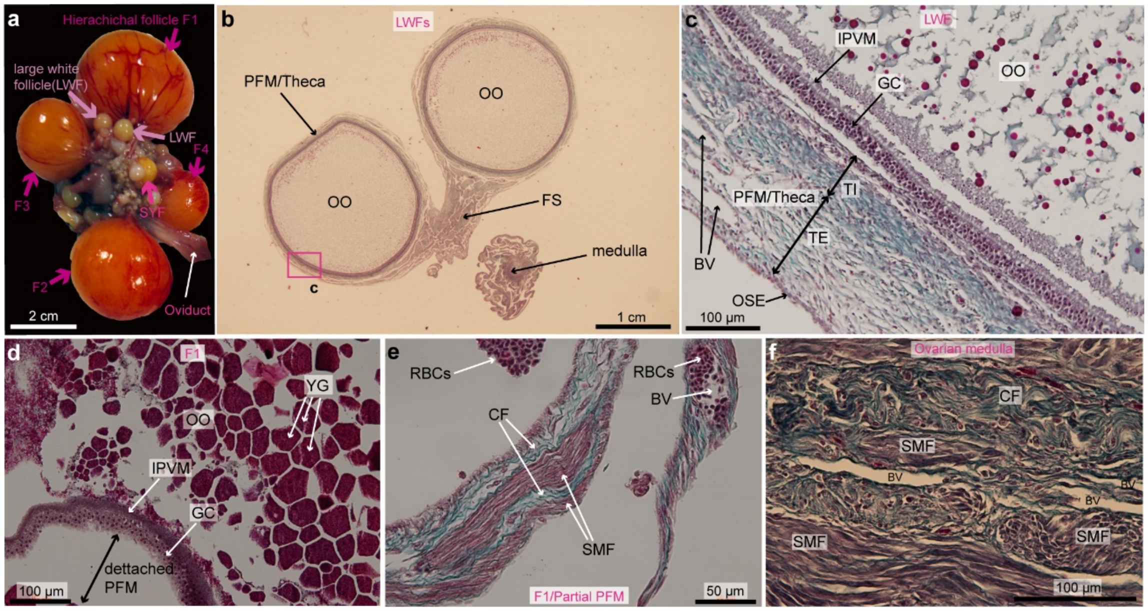

Supplementary Figure 1. Gross morphology (a) and histology (b-f) of ovarian follicles in an extant hen.

(a) Photograph of functional (left) ovary after dissection showing immature follicles: large white follicles (LWF) and a small yellow follicle (SYF); and much more mature follicles that have entered a hierarchy, called hierarchical follicles (F1, F2, F3, F4). F1 is the follicle that is supposed to be ovulated on the next day. (b) Paraffin section of two large white follicles. (c) Close-up of the pink rectangle in B, showing all the different tissues making the follicle: the oocyte, inner perivitelline membrane, granulosa cells and the ovarian perifollicular membrane (also called the theca, double-headed arrow). (d) Paraffin slide of the yolk-filled oocyte and the inner-perivitelline membrane of the hierarchical follicle F1 (the closest to ovulation) (d). The perifollicular membrane of F1 detached during sample processing and is shown in E. The double-headed arrow shows where the perifollicular membrane should have been had it not detached during sample preparation and processing. (e) Close-up on the detached perifollicular membrane of F1, showing collagen fibers and smooth muscle fibers organized into a contractile structure called the chordae. There are much more smooth muscle fibers in the perifollicular membrane of hierarchical follicles (corresponding to the late formation of the chordae) that in the much ‘younger’ large white follicles. (f) Section through the ovarian medulla. Note that the perifollicular membrane of immature follicles, mature follicles and the ovarian medulla have the same main tissue types (i.e., blood vessels, collagen fibers, and smooth muscle fibers), but what changes is simply their proportions and organization. All slides were stained with a modified Masson’s trichrome to reveal muscle fibers in pink and collagen fibers in green.

Abbreviations: BV, blood vessel; CF, collagen fibers; FS, follicular stalk of the ovary; GC, granulosa cells; IPVM, inner perivitelline membrane; OO, oocyte; OSE, ovarian surface epithelium; PFM, perifollicular membrane; RBCs, red blood cells; SMF, smooth muscle fibers; SYF, small yellow follicle; TI, theca interna; TE, theca externa; YG, yolk granule.

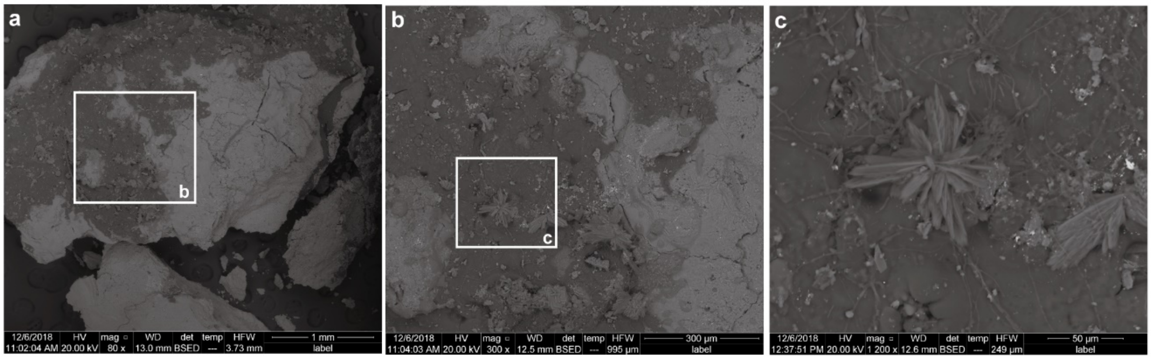

Supplementary Figure 2. Surface SEM on the first fragment of STM10-12 prior to ground-sectioning.

(a) General view of the fragment. (b) Close-up of the square in (a). (c) Close-up of the square in (b). These images show crystals, on top of a dark material partially covering a much lighter material. The dark material is a consolidant that was applied to the fossil prior to the start of this study.

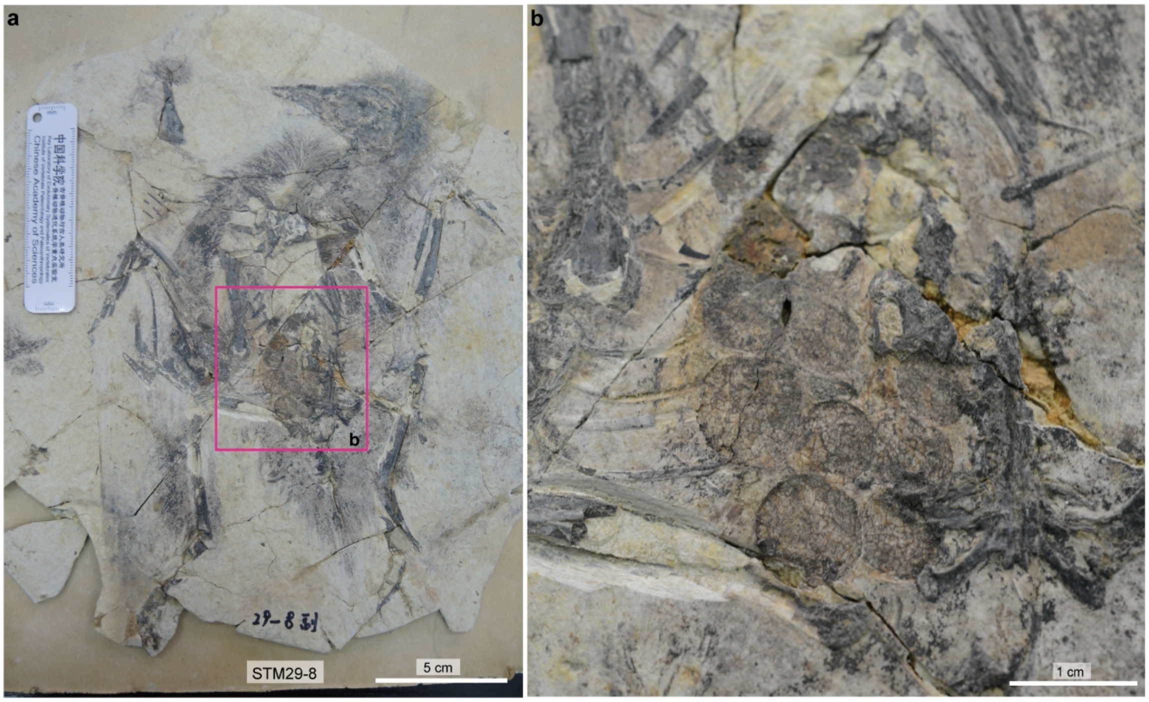

Supplementary Figure 3. Photographs of the enantiornithine indet. STM29-8 and its purported ovarian follicles.

(a) Photograph of the entire specimen. (b) Close-up of the pink rectangle in A showing the agglomerated ovarian follicles. Zheng et al., 2013 originally hypothesized that these follicles had imprints of blood vessels from the perifollicular membrane.

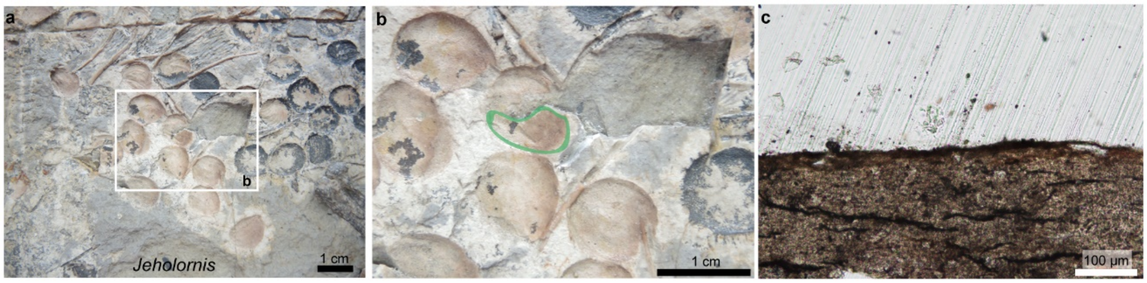

Supplementary Figure 4. Photographs of ingested seeds in *Jeholornis* IVPP V13274, and cross-section of a fragment.

(a) Seeds preserved in the stomach of a specimen of *Jeholornis* (IVPP13274). (b) close-up in A. A fragment of seed was extracted (green outline) and analyzed histologically. (c) Cross section of the seed fragment seen under the transmitted light. Only sediment is observed, with a slight brown stain on the surface. This cross section was also scrutinized at much higher magnification under the scanning electron microscope but again only sediment was seen (data not shown). This revealed that in this specimen, the 'seeds' have no preserved tissues and are only brown seed impressions.

Supplementary Table 1. Additional data concerning all other Jehol specimens reported with follicles.

Taxon (Aves)	Specimen no.	Femur length (mm)	Follicle diameter range (mm) and/or average diameter (mm)	Gross morphology-Surface texture	Microscopic examinations	Other apparent soft-tissues preserved on the specimen?	References
Jeholornis prima	STM2-51	88.072	6.70-8.79; average is 7.65	Surface permineralized by a dark macrocrystalline mineral in the slab. (Pale pink impressions with some small crystals of that black mineral in the counterslab).	None	no obvious preservation, and overall poor quality of bone preservation at the gross morphological level	Zheng et al., 2013 [1] (See their Fig. S2)
Eoconfuciusornis	STM7-144	25.4	3.4-5.8	The follicles preserved as slightly three-dimensional voids. Ventral surface of the void bears a circular micro-pattern, hypothesized to be the result of authigenic mineral growth and not a natural morphology of the follicles.	Surface SEM	exceptional soft tissue preservation such as feathers and wing patagium	Zheng et al., 2017 [2]
Enantiornithes indet.	STM29-8	40.248	5.83-8.83; average is 6.86	Surfaces with a black carbonization, paired with an uneven lattice structure of fibres in the slab. (Impressions in the counterblab).	The 'uneven lattice structure of fibers' were hypothesized to be imprints of the blood vessels from the highly vascularized perifollicular membrane (Zheng et al., 2013).	feather impressions, but overall poor quality of bone preservation at the gross morphological level	Zheng et al., 2013 [1] (See their Fig. S5)
Enantiornithes indet.	STM10-45	34.9	6.72-8.78; average is 7.56	Surface with small, black circular spots of mineralization; the roundness of these structures suggested that preservation of the follicles in this specimen may have been bacterially mediated.	none	no obvious preservation, and overall poor quality of bone preservation at the gross morphological level	Zheng et al., 2013 [1] (See their Fig. S7)
Enantiornithes indet.	STM10-12	_	average is 7.7	This study: Surface with pale pink impressions, with a cracked surface, and some areas with a brown/ dark orange tint, on both the slab and counterslab. The slab has more broken rib fragments that the counterslab.	This study (Fragments of the counterslab- see results section): Standard ground-sectioning, SEM-EDS; paleohistochemistry	preserves some body feathers and overall poor quality of bone preservation at the gross morphological level	This study and O'Connor et al., 2013 [3]
Enantiornithes indet.	STM10-4	38.7	average is 7.15	Grainy, uneven, black texture.	None	some feathers (poorly preserved), overall very poor preservation of the skeleton.	O'Connor et al., 2013 [3]
Enantiornithes indet.	STM11-121	37.5	average is 6.8	Grainy, uneven, black texture.	None	no obvious preservation of soft-tissues, skeleton poorly preserved and lots of reddish (iron?) staining near and surrounding the skeleton	O'Connor et al., 2013 [3]
Enantiornithes indet.	STM11-212	~32-35	average is 5.4	Grainy, uneven, black texture.	None	preserves some feathers and potential skin traces, skeleton moderately preserved.	O'Connor et al., 2013 [3]
Linyiornis amoena	STM11-80	37.7	average is 6.5	Surface described as black carbonized soft-tissue remains.	None	no other obvious preservation of soft-tissues, skeleton well preserved in three-dimensions (due to calcite crystals filling the medullary cavity)	O'Connor et al., 2013 [3], Wang et al., 2016 [4]

Supplementary Methods

More detailed protocol for paraffin sections.

After demineralization of the agar-embedded fossil tissues and the fixation in Neutral Buffered Formalin of the extant tissues, the following steps were followed: all samples were subjected to routine dehydration via sequential incubations in 70%, 80%, 90%, 95%, and 100% EtOH for ~ 1 hour each. The tissue blocks were placed in two additional 100% EtOH for 1 hour each to ensure complete dehydration, followed by three 30 min incubations in xylene. Tissues were infiltrated with melted paraffin wax for 30 min each and embedded manually (Paraplast Plus EMS Cat#19216).

Blocks were cooled down in an ice bath and cut on a microtome (Leica Biosystems RM2265), placed in a warm water bath at 44°C with section adhesive (Tissue-Grip, StatLab), mounted on charged slides (Superfrost Plus, Fisher Scientific), then dried in an oven at 60°C for one hour, or overnight at room temperature.

Paraffin sections were then deparaffinized with xylene, dehydrated through a graded EtOH series (70%, 80%, 90%, 95%, and 100% EtOH for 1 min each), and stained with a modified Masson's trichrome protocol[5]. They were stained for 10 min with Mayer's acid hematoxylin (Sigma MSH-32), rinsed in deionized water for 1 min, stained with Xylidine Ponceau/Acid Fuschin for 2 min (equal volumes of 0.5% xylidine ponceau 2R CI no. 16150 in 1% acetic acid and 0.5% acid fuchsin CI no. 42685 in 1% acetic acid), rinsed for 10 secs in deionized water, stained for 4 min with 1% phosphomolybdic acid, rinsed for 10 secs in deionized water, stained with light green for 90 secs (2% light green CI 42095 in 2% citric acid, diluted 1 : 10 with deionized water prior to use) and rinsed in deionized water. Sections were then dipped twice in 100% EtOH for 10 s, cleared in xylene for 4 minutes, and cover-slipped with Permount (Fisher Scientific).

Technical notes on the choice of extant follicles for histology:

Extant female birds have different types and sizes of follicles in their ovary[6,7]. During the dissection of the hen (Fig. S1), we sampled immature follicles called 'large white follicles' (LWF) based on their size and color (Fig. S1a, according to [6,7]). They were processed and embedded whole into a mold (Fig. S1b-c). We also sectioned part of the perifollicular membrane and some of its attached yolk of a follicle closest to ovulation (called 'hierarchical follicle F1', Fig. S1a). Because F1 is so large, it did not fit in the mold and it was not feasible to section it whole (Fig. S1d-f) like the other LWFs (Fig S1b).

Contamination Issues

Demineralization and dehydration steps between the fossil and extant tissues were performed in two separate rooms, using different equipment at IVPP. All samples were cut on the same microtome, but not using the same microtome blades between the fossils and the extant blocks of tissues. We did not use the same water for the water-bath (right after the microtome cut) between extant and fossil tissues either. Deparaffinization, dehydration and histochemical staining for the fossil and the extant tissues were made in two different sets of solutions and staining dishes, which did not come in contact with each other anytime during the process. By doing this, we avoided any extant tissue to 'land' on a glass slide with fossil tissues (and *vice versa*).

SUPPLEMENTARY REFERENCES

1 Zheng, X. *et al.* Preservation of ovarian follicles reveals early evolution of avian reproductive behaviour. *Nature* **495**, 507 (2013).

2 Zheng, X. *et al.* Exceptional preservation of soft tissue in a new specimen of Eoconfuciusornis and its biological implications. *National Science Review* **4**, 441-452 (2017).

3 O'Connor, J. K., Zheng, X., Wang, X., Wang, Y. & Zhou, Z. Ovarian follicles shed new light on dinosaur reproduction during the transition towards birds. *National Science Review* **1**, 15-17 (2013).

4 Wang, Y. *et al.* A new Jehol enantiornithine bird with three-dimensional preservation and ovarian follicles. *Journal of Vertebrate Paleontology* **36**, e1054496 (2016).

5 Witten, P. E. & Hall, B. K. Seasonal changes in the lower jaw skeleton in male Atlantic salmon (Salmo salar L.): remodelling and regression of the kype after spawning. *Journal of Anatomy* **203**, 435-450 (2003).

6 Apperson, K., Bird, K., Cherian, G. & Löhr, C. Histology of the Ovary of the Laying Hen (Gallus domesticus). *Veterinary sciences* **4**, 66 (2017).

7 Manickam, S. R. & Kundu, A. Regulation of ovarian follicular Atresia through apoptotic process in Japanese quail (Coturnix coturnix japonica).
