# Supplementary material for: Confirmation of ovarian follicles in an enantiornithine (Aves) from the Jehol biota using soft tissue analyses
Source: Commun Biol. 2020 Jul 28;3:399. doi: 10.1038/s42003-020-01131-9 (PMC7387556; doi:10.1038/s42003-020-01131-9)
Supplement: Supplementary file 2 — Reporting Summary [file 42003_2020_1131_MOESM2_ESM.pdf]

nature research

Corresponding author(s): Alida Bailleul

Last updated by author(s): Jul 7, 2020

Reporting Summary

Nature Research wishes to improve the reproducibility of the work that we publish. This form provides structure for consistency and transparency in reporting. For further information on Nature Research policies, see our Editorial Policies and the Editorial Policy Checklist.

Statistics

For all statistical analyses, confirm that the following items are present in the figure legend, table legend, main text, or Methods section.

n/a	Confirmed	
☒	☐	The exact sample size (*n*) for each experimental group/condition, given as a discrete number and unit of measurement
☒	☐	A statement on whether measurements were taken from distinct samples or whether the same sample was measured repeatedly
☒	☐	The statistical test(s) used AND whether they are one- or two-sided<br>*Only common tests should be described solely by name; describe more complex techniques in the Methods section.*
☒	☐	A description of all covariates tested
☒	☐	A description of any assumptions or corrections, such as tests of normality and adjustment for multiple comparisons
☒	☐	A full description of the statistical parameters including central tendency (e.g. means) or other basic estimates (e.g. regression coefficient) AND variation (e.g. standard deviation) or associated estimates of uncertainty (e.g. confidence intervals)
☒	☐	For null hypothesis testing, the test statistic (e.g. *F*, *t*, *r*) with confidence intervals, effect sizes, degrees of freedom and *P* value noted<br>*Give P values as exact values whenever suitable.*
☒	☐	For Bayesian analysis, information on the choice of priors and Markov chain Monte Carlo settings
☒	☐	For hierarchical and complex designs, identification of the appropriate level for tests and full reporting of outcomes
☒	☐	Estimates of effect sizes (e.g. Cohen's *d*, Pearson's *r*), indicating how they were calculated

Our web collection on statistics for biologists contains articles on many of the points above.

Software and code

Policy information about availability of computer code

Data collection	We did not use computer codes to collect the data.
Data analysis	We did not use computer codes to analyze the data.

For manuscripts utilizing custom algorithms or software that are central to the research but not yet described in published literature, software must be made available to editors and reviewers. We strongly encourage code deposition in a community repository (e.g. GitHub). See the Nature Research guidelines for submitting code & software for further information.

Data

Policy information about availability of data

All manuscripts must include a data availability statement. This statement should provide the following information, where applicable:

- Accession codes, unique identifiers, or web links for publicly available datasets
- A list of figures that have associated raw data
- A description of any restrictions on data availability

STM10-12 is reposited at the Shandong Tianyu Museum of Nature in Linyi City. Ground-sections and paraffin sections of STM10-12 fragments are currently reposited at the Institute of Vertebrate Paleontology and Paleoanthropology in Beijing. All data are available upon reasonable request.

Field-specific reporting

Please select the one below that is the best fit for your research. If you are not sure, read the appropriate sections before making your selection.

☐ Life sciences ☐ Behavioural & social sciences ☒ Ecological, evolutionary & environmental sciences

For a reference copy of the document with all sections, see nature.com/documents/nr-reporting-summary-flat.pdf

Ecological, evolutionary & environmental sciences study design

All studies must disclose on these points even when the disclosure is negative.

Study description	This study involves a fossil specimen that was already described in another study, and one specimen of cadaveric hen that was obtained legally and commercially. All categories below are technically N/A but will be clearly explained.
Research sample	STM10-12: a fossil enantiornithine; and a cadaveric hen (Gallus domesticus).
Sampling strategy	The sample size is too small to perform any statistical analysis and therefore we have no true 'sampling strategy'.
Data collection	We sampled fragments of the two specimens to perform histological analyses.
Timing and spatial scale	Timing and spatial scale is irrelevant in this histological study.
Data exclusions	No data were excluded.
Reproducibility	Reproducibility is feasible, histological sampling can be performed on additional specimens, but it was not necessary in this study.
Randomization	Randomization is not relevant in this study, because we simply compare ovarian histology of a fossil to bird to that of an extant bird.
Blinding	Blinding was not relevant in this study, because we simply compare ovarian histology of a fossil to bird to that of an extant bird.

Did the study involve field work? ☐ Yes ☒ No

Reporting for specific materials, systems and methods

We require information from authors about some types of materials, experimental systems and methods used in many studies. Here, indicate whether each material, system or method listed is relevant to your study. If you are not sure if a list item applies to your research, read the appropriate section before selecting a response.

Materials & experimental systems

n/a	Involved in the study	
☒	☐	Antibodies
☒	☐	Eukaryotic cell lines
☐	☒	Palaeontology and archaeology
☒	☐	Animals and other organisms
☒	☐	Human research participants
☒	☐	Clinical data
☒	☐	Dual use research of concern

Methods

n/a	Involved in the study	
☒	☐	ChIP-seq
☒	☐	Flow cytometry
☒	☐	MRI-based neuroimaging

Palaeontology and Archaeology

Specimen provenance	Specimen STM10-12 is from the Jehol biota of Northeastern China, already described in a previous study (O'Connor et al., 2013; cited in our current paper)
Specimen deposition	STM10-12 is reposited at the Shandong Tianyu Museum of Nature in Linyi City
Dating methods	No new dates were provided - We cite a paper with more precise data.

☒ Tick this box to confirm that the raw and calibrated dates are available in the paper or in Supplementary Information.

Ethics oversight	No ethical approval nor guidance was necessary. The fossil specimen does not require any ethics approval for sampling, and the chicken was obtained from a farm that legally sells chickens for human consumption.

Note that full information on the approval of the study protocol must also be provided in the manuscript.
